# 

**Published:** 1906-08

**Authors:** 


### CONTENTS.

ORIGINAL COMMUNICATIONS—

THE INTESTINAL TOXEMIAS OF CHILDREN. By L. B. Clark, M.D., Atlanta, Ga. .... 289
A PLEA FOR MORE LABORATORY WORK. By E. C. Thrash, M.D., Atlanta, Ga. .... 294
LIFE INSURANCE EXAMINATIONS. By Wilson Williams, Binghampton, N. Y. .... 298
THE ADMINISTRATION OF DRUGS IN CONJUNCTION WITH PHYSICAL METHODS IN THE TREATMENT OF DISEASE. By W. T. Rushin, M.D., Macon, Ga. .... 301
THE SPECIALIST'S POINT OF VIEW. By Ross P. Cox, Rome, Ga. .... 302

CORRESPONDENCE .... 306

EDITORIAL NOTES AND COMMENTS—

LIFE INSURANCE EXAMINING .... 310

NEWS AND NOTES .... 314

BOOK REVIEWS—

CONSUMPTION—ITS RELATION TO MAN AND HIS CIVILIZATION—ITS PREVENTION AND CURE. (Huber) .... 318
PROGRESSIVE MEDICINE. (Hare.) .... 319
A NURSE'S HAND-BOOK OF MEDICINE. (Henry.) .... 319
A TREATISE ON SURGERY. (Fowler.) .... 320
GOLDEN RULES OF SURGERY. (Bernays.) .... 320
SURGICAL SUGGESTIONS. (Br[illegible]ckner.) .... 321
PRELIMINARY ANNOUNCEMENT .... 321

CONTENTS CONTINUED .... X

